# Reported Behavioral Patterns and Concern Surrounding Well Water Testing for Arsenic in Midwestern Homeowners with Children

**DOI:** 10.3390/ijerph22040504

**Published:** 2025-03-26

**Authors:** Dominika A. Jegen, Julie Maxson, Natalie Averkamp, Rachael Passmore, Jessica L. Sosso, Stephen K. Stacey, Tom D. Thacher

**Affiliations:** 1 Department of Family Medicine, Mayo Clinic, 200 First St SW, Rochester, MN 55905, USA; maxson.julie@mayo.edu (J.M.); passmore.rachael@mayo.edu (R.P.); thacher.thomas@mayo.edu (T.D.T.); 2 Department of Quantitative Health Sciences, Mayo Clinic, 200 First St SW, Rochester, MN 55905, USA; averkamp.natalie@mayo.edu; 3 Department of Family Medicine, Mayo Clinic Health System, 310 W. Main St., Sparta, WI 54656, USA; sosso.jessica@mayo.edu; 4 Department of Family Medicine, Mayo Clinic Health System, 800 West Ave. S., La Crosse, WI 54601, USA; stacey.stephen@mayo.edu

**Keywords:** arsenic, heavy metals, metalloids, pediatrics, public health, water quality, water testing, well water

## Abstract

Toxins from ingested water can significantly affect overall physical health in children and adults. In the United States, domestic water wells are not commonly tested for any heavy metal contaminants. It is well-known that chronic arsenic ingestion from water is linked to serious health effects. We surveyed patients at our academic institution via emailed questionnaires in 2023 to determine whether those with children living at home reported different patterns of behavior around well water testing as compared to those without. Survey data were collected from 8994 respondents in the U.S. Midwest who reported using residential well water. Results were used to evaluate the influence of children in the home on testing behaviors, and secondarily, whether parental demographics affected testing frequency. Respondents with children at home did not report increased testing frequency compared to those without. In parents who did report testing, having more children, being younger, and living in Wisconsin were associated with an increased frequency. Parental gender, race, and ethnicity did not correlate with testing behaviors. A total of 70% of respondents did not feel concerned about their water safety and 85% were not concerned about arsenic specifically in their water. Increased risk of toxicity to children from arsenic does not appear to influence reported well water testing behavior among parents.

## 1. Introduction

Environmental toxins such as heavy metals are known to be present in topsoil, water aquifers, and crops used for sustenance worldwide [1,2]. In the United States, domestic well water is a known risk for heavy metal exposure as it is not under public health or governmental oversight and has little regulation, as opposed to city well water for which testing is carried out [3,4,5].

Arsenic specifically is a concern in Midwestern water sources primarily because of naturally occurring geological deposits and agricultural practices that contribute to contamination. Some Midwestern states, such as Minnesota, Wisconsin, Michigan, Iowa, Nebraska, and North and South Dakota encompass areas where groundwater arsenic levels exceed the Environmental Protection Agency’s maximum contaminant levels. Pesticides and fertilizers used in farming may contribute to contamination. Livestock feed additives containing arsenic were previously used, and hence resulting runoff from agricultural areas can lead to arsenic exposure. Industrial activities such as mining, manufacturing, and coal incineration also contribute to arsenic levels in groundwater [6,7]. While routinely referred to as a heavy metal, arsenic is technically considered a metalloid [8].

When used as a source of potable water and for daily household activities, water contaminated with arsenic impacts human health through pathophysiological processes causing diseases such as diabetes, hypertension, coronary artery disease, neuropathy and various cancers [9,10,11,12,13,14,15,16,17,18]. Young children are at the greatest risk of illness due to the toxic effects of well water contaminants such as arsenic, including reduced infant birth weight, newborn cleft lip and palate [19,20], and increased risk of fetal spina bifida [21]. Older children exposed to water contaminated with arsenic are also more likely to be underweight [22]. The American Heart Association published a statement in 2023 indicating that for all citizens, “chronic exposure to low levels of lead, cadmium and arsenic through commonly used household items, air, water, soil and food is associated with an increased risk of cardiovascular disease”, increasing coronary artery disease, myocardial infarction, and stroke risk [14]. Research performed by Farzan et al. in Bangladeshi adolescents further suggests that chronic arsenic exposure may be related to endothelial dysfunction, especially among females, and is hypothesized to increase risk of later adverse cardiovascular health events [23].

Both prenatal and childhood exposure to arsenic can negatively affect cognitive ability [24,25]. This can be seen as soon as the child begins school [26] and may continue to affect neurodevelopmental scores in their teenage years [27]. As the individual further ages, existing literature has demonstrated that higher arsenic levels in environmental samples are associated with a higher risk of neurodegenerative diseases such as Alzheimer’s and Parkinson’s in those living in affected areas [28]. Conversely, lower levels of arsenic in domestic drinking water are associated with increased perceptual reasoning, working memory, and verbal comprehension scores in children [29]. In Bangladesh, where it is estimated that at least 35 million people are exposed to arsenic through drinking water, the installation of safer community water wells was associated with improvements in pediatric memory scores, though was not associated with an improvement in intelligence quotient (IQ) [30]. Interestingly, low arsenic is also associated with increased levels of maternal IQ and maternal educational attainment, possibly reflecting protective parental behaviors such as water filter use and choice of residential location [29].

Recently published literature has highlighted the need for clinicians to screen for dangerous exposures from pediatric patients’ rural environments, “especially from well water contaminants, wood stove smoke, and various agricultural toxins” [31]. The “contaminants found in these exposures are known to adversely cause respiratory, neurodevelopmental, cardiometabolic, and carcinogenic effects, and the authors recommend that rural pediatric clinicians screen for these environmental exposures, and they provide tools and resources related to testing, mitigation, and medical monitoring” [31,32]. As Cradock et al. write, “drinking water free of lead, nitrates and arsenic is vital for infant and young children’s health” given its well-documented and far-reaching negative pathophysiologic processes [33].

Currently, 13% of Americans, or 43 million people, rely on private wells for drinking water [6,12]. In many areas of the Midwest, private well water use is even more prevalent, with 10% use in Iowa [34], 22% use in Minnesota [35], and almost 25% in Wisconsin [36]. In 2009, a policy statement from the American Academy of Pediatrics provided algorithms for annual inspection, testing, and remediation of wells providing drinking water for children’s use in homes, daycares, and schools [3,4]. It was again reaffirmed in 2013 and 2019 given the importance of safe water for children’s development.

However, population surveys conducted by the Minnesota Department of Health have shown that fewer than 20% of private well owners evaluate their wells at the recommended frequency [37]. A recent study in Iowa found that approximately 40% of households do not regularly test, treat, or avoid ingesting their well water, suggesting that pollution exposure may be widespread among this Midwestern population [38]. When previously asked as to why they do not test their water, well owners report not being concerned about arsenic, not being sure what to do or whom to contact, feeling treatment options are expensive or too difficult to maintain, and not having enough time [37,39].

While children are at the highest risk of illness due to contamination of well water with toxins such as arsenic, they are dependent on caregivers to maintain a safe drinking environment. Parental knowledge of well contamination risks and testing results are important factors in promoting self-reported well stewardship behavior [40,41]. In order to evaluate whether people with children in the home are more inclined to follow testing recommendations, we performed a cross-sectional survey of adults in the upper Midwest region of the United States. We compared reported well water testing behaviors in respondents with children as compared to those without children at home. While this study does not report objective arsenic levels in well water or their impact on health outcomes as other studies have already done previously [27,29,30,42,43], it describes parents’ self-reported attitudes towards testing behaviors and their concerns regarding potential arsenic contamination of their well water.

## 2. Materials and Methods

### 2.1. Study Overview

This study was a cross-sectional descriptive survey of actively paneled patients at the Mayo Clinic and the Mayo Clinic Health System in the Midwest of the United States, comprising the states of Minnesota, Wisconsin, and Iowa. This includes the Mayo Clinic and more than forty community-based hospitals and clinics across southern Minnesota, western Wisconsin, and northern Iowa. The study was reviewed and determined to be exempt from the requirement for Institutional Review Board (IRB) approval by the Mayo Clinic. Data were collected from patients who self-identified as having residential well water. The survey methods have been previously published by our research team [10].

Survey responses were kept confidential and de-identified before being analyzed or shared with study investigators. Non-responders received two follow-up reminders to complete the survey. The collected data were analyzed to assess the awareness of patients regarding the health effects of arsenic toxicity from well water, the necessity of routine well water testing, and whether they desired more information about the risks of chronic arsenic exposure.

### 2.2. Study Methods

Emails containing the survey and a consent script were sent to all adult patients over the age of 18 years with an email address who were paneled at our institution at the time of the survey. All other patients under the age of 18 and/or without an email address were excluded. The email script served as the oral consent and patients were told if they agreed to participate, they should click the survey link to complete it. Active patient email addresses fitting our study criteria were obtained from existing clinical data repositories which are used for institutional research. A total of 279,798 patient email addresses were initially included. Those respondents and families with a duplicate email address only received one survey (resulting in the removal of 22,367 email addresses).

**Figure 1:**
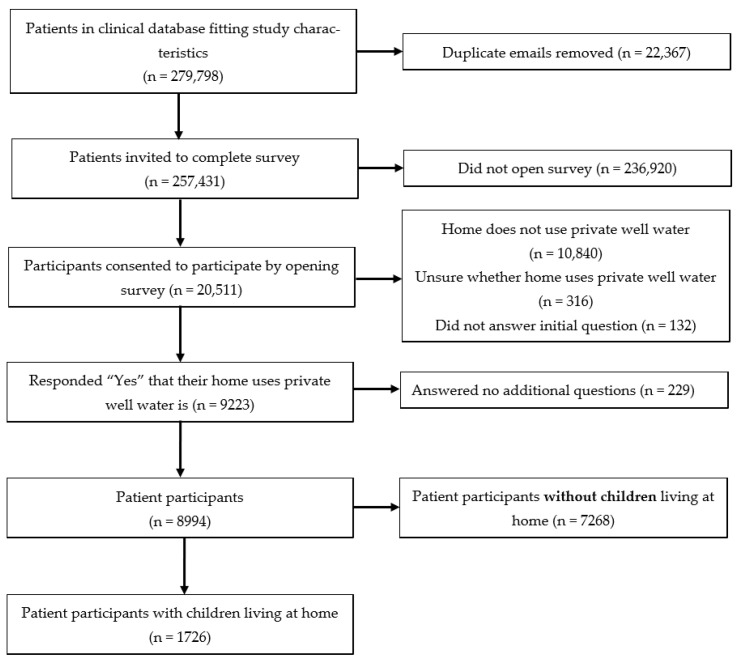
Study protocol for inclusion of patients with and without children living at home.

Survey sampling is illustrated in Figure 1 and was completed over six weeks in September and October of 2023. In brief, the survey took three minutes to complete and included questions pertaining to frequency of well water testing, respondent’s concern for the health effects of well water, and concern for arsenic specifically, as well as demographic factors such as state of residence, number of children in the home, age, gender, race, and ethnicity. Survey responses were kept confidential and de-identified prior to analysis or report to study investigators. Nonresponders received two follow-up reminders to complete the survey. Data were then analyzed to differentiate between participants with children as compared to those without children regarding water-testing behaviors, awareness of health effects from well water, and concern about their water safety with regards to arsenic contamination (Supplementary File S1).

For those with children, we also assessed whether number of children, parental age, state of residence, gender, or ethnicity played a role in testing frequency and concern regarding water contamination with arsenic. For simplicity, we refer to adults with children living at home as “parents”. Respondents may have been other family members who house or serve as caregivers, such as grandparents.

### 2.3. Statistical Analysis

Ordinal logistic regression and chi-squared tests were used to assess the relationship between survey answers and demographics of respondents. The proportional hazards assumption of the ordinal logistic regression models was assessed visually by plotting the predicted coefficients without the parallel slopes assumption and by comparing the Akaike Information Criterion (AIC) of each model to a multinomial model. Odds ratio outcomes were aimed at the likelihood of testing more frequently, not just the likelihood of testing once. R Statistical software (version 4.3.2, R Core Team 2023) was used to develop reports.

## 3. Results

Of the 257,431 participants that were invited to take the survey, 20,511 opened the survey for an 8% response rate (Figure 1). A total of 9223, or nearly half (44%) of respondents, reported primarily using private well water in their home [10]. After excluding 229 individuals that did not answer any additional survey questions, 8994 participants completed the remainder of the survey. Of these, 1726 indicated they had one or more children living in their home. The respondents were mostly female, White, older, and located in Wisconsin.

**Figure 2:**
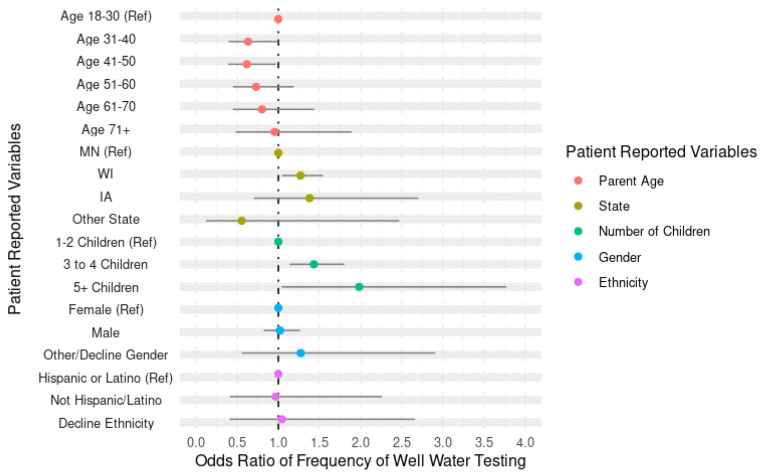
Odds ratio of the frequency of well water testing by parents with children at home as compared to patient-reported variables.

When compared to those without any children at home, those with children at home did not differ statistically in their reported frequency of well water testing (*p*-value 0.144; Table 1). A secondary analysis was then completed to assess only the participants with children at home. When assessing these respondents’ testing frequency, with possible options ranging from never to annually, those aged 31–40 years and aged 41–50 years appear less likely to test frequently than the reference group aged 18–30 years (Figure 2).

Households with more children were more likely to report frequent water testing. Parents with three to four children (13.1% tested annually) and those with five or more children (29.3% tested annually) were more likely to test their water regularly compared to parents with one to two children (10.6% tested annually) (Table 2).

When assessing whether patients with children at home had ever tested their water, the older they were, the higher the likelihood that they had tested at least once in the past (Table 3). There were no associations between the frequency of worrying about the health effects of well water, worrying about arsenic in well water, or interest in information about testing for arsenic in well water. A total of 70% of respondents stated that they never or rarely worried about the safety of their well water, and 85% never or rarely worried about arsenic specifically.

Respondents in Wisconsin were more likely to test than those in Minnesota or Iowa and testing frequency was not associated with reported parental gender, race, or ethnicity (Figure 2).

## 4. Discussion

Arsenic exposure in childhood is a major risk for the development of comorbidities as the individual ages [27,44]. It is associated with higher serum glucose and the development of diabetes in affected children [45] with this risk continuing even as they become adults [44]. Children in Chile who were exposed to exceedingly high arsenic levels had an increased risk of bronchiectasis that correlated with age at first exposure. This risk continued to be seen even thirty to forty years after exposure [46]. This same cohort in Chile showed increased associations with laryngeal, lung, and bladder cancers [46]. In Bangladesh, chronic exposure was linked to a higher mortality rate even after the children became adults [42]. These findings suggest that interventions targeting early-life arsenic exposure could have major impacts in reducing long-term morbidity and mortality [47].

This study illustrates that survey participants who have children at home do not test their well water any more frequently than those without children at home. Well water safety and concern about arsenic was low among all respondents. In parents who do test their water, younger parents test more frequently than those who are older, indicating that they are presumably more concerned about well water safety, have greater awareness of testing options, or are more comfortable with testing processes. This is in keeping with accumulating evidence indicating that environmental health concerns have increased in preceding decades but suggests an ongoing generational gap [48,49]. Research has shown that younger respondents typically display higher levels of environmental concern as compared to their older counterparts [47,48,49]. A 2017 study of Wisconsin private well users additionally found that living in particular geographic regions of the state, as well as having an increased annual income, were the most significant predictors of well water testing and filtration [39]. Both variables could potentially account for the findings seen in our study, although these specific data were not collected from our study population.

The older age groups do report testing at least once at a higher proportion than younger age groups, which is intuitive given that the older someone is, the more likely it is that they have tested their water at least once in their lifetime, or perhaps they do not expect findings to change on retesting. This also explains why there is no association between children at home and testing frequency, as older individuals are less likely to have children in the home. The younger respondents constituted a smaller proportion of this cohort and had more missing data, which may affect this analysis and conclusions.

We found that parents in Wisconsin test more frequently than those in Iowa or Minnesota, in keeping with our previously published findings on the entire sampled population [10]. This may result from the fact that Wisconsin residents rely on private wells more than residents of these other states (25% versus 13% nationally) and may be more familiar with the process of doing so [36]. Current state recommendations are to test annually in all three states, although actual laws are less stringent. For example, Minnesotans are only required to test well water for arsenic once at the time of initial construction [35]. Hence, even if a well is tested repeatedly, there is no requirement to test for arsenic specifically.

Lastly, respondents’ gender, race, and ethnicity did not correlate with testing behaviors or concern about water safety. The explanation for this is related to the small population of non-White patients residing in the upper Midwest. These groups were not adequately sampled in this survey approach as over 90% of our sample self-identified as White. A targeted survey or focus group would better assess testing behaviors in minority populations.

One notable limitation of the study is the absence of direct measurement or verification of actual arsenic levels in respondents’ water. Although we attempted to correlate well water testing behaviors with the other variables described, there was no direct measurement or confirmation regarding actual arsenic analyses. These results rely on participant report with regards to testing frequency and there is no verification that respondents’ wells were tested for arsenic specifically. Patients may have also been testing for other contaminants and may have seen arsenic as a byproduct of that concern or as an element with less importance as compared to bacteria and nitrates, for example [39]. Study limitations also include a low response rate of 8%. Patients at our institution are frequently requested to participate in research, and as such we suspect that participation fatigue contributed. Respondents were self-selected and non-randomized in terms of opening the survey request and its completion, which introduced participation bias and response bias. Given that patients likely know that safe water is important for their children, conformity bias cannot be ruled out. In order to keep our survey succinct and to improve response rate by its brevity, we elected to only include the questions that were paramount for us to explore. For this reason, we did not ask about reasons for carrying out (or foregoing) well water testing. A survey conducted earlier in Minnesota did ask these questions, however [37]. Lastly, we cannot determine cause and effect from this cross-sectional analysis.

## 5. Conclusions

When compared to those without any children, patients with children at home did not differ statistically in their reported frequency of well water testing. Of those parents who do test, older participants aged 31–50 years appear less likely to test than the reference group aged 18–30 years. Having more children at home was associated with a higher likelihood of testing more frequently. Those living in Wisconsin are more likely to test than those in Minnesota or Iowa. There were no associations between testing frequency and parental gender, race, or ethnicity.

Further studies are needed to determine the most efficient way to identify children who use well water and to educate their parents on the importance of regular testing to prevent serious long-term sequelae of contaminated water ingestion. In the meantime, all clinicians including pediatricians, family physicians, physician assistants and pediatric nurse practitioners should advise parents using well water about the importance of testing for toxins in their potable water, particularly in rural areas.

## Figures and Tables

**Table 1:** The presence of children or no children at home versus frequency of well water testing.

	Never (*n* = 1923)	Once (*n* = 1415)	Every 6–10 Years (*n* = 1214)	Every 2–5 Years (*n* = 1857)	Once a Year (*n*= 1016)	Missing (*n* = 1569)	Total (*n* = 8994)	*p* Value
								0.144
Has 1+ Children at Home	374 (21.7%)	279 (16.2%)	200 (11.6%)	348 (20.2%)	201 (11.6%)	324 (18.8%)	1726 (100.0%)	
Has No Children at Home	1523 (21.4%)	1122 (15.7%)	1006 (14.1%)	1491 (20.9%)	793 (11.1%)	1198 (16.8%)	7133 (100.0%)	
Number Missing	26	14	8	18	22	47	135	

**Table 2:** In respondents with children at home, the number of children versus frequency of well water testing.

How Many Children Live in Your Home?	Never (*n* = 374)	Once (*n* = 279)	Every 6–10 Years (*n*= 200)	Every 2–5 Years (*n*= 348)	Once a Year (*n* = 201)	Missing (*n* = 324)	Total (*n* = 1726)
1 to 2	295 (22.7%)	213 (16.4%)	152 (11.7%)	246 (19.0%)	138 (10.6%)	253 (19.5%)	1297 (100.0%)
3 to 4	70 (18.0%)	62 (16.0%)	46 (11.9%)	91 (23.5%)	51 (13.1%)	68 (17.5%)	388 (100.0%)
5+	9 (22.0%)	4 (9.8%)	2 (4.9%)	11 (26.8%)	12 (29.3%)	3 (7.3%)	41 (100.0%)

**Table 3:** In respondents with children at home, the respondent’s age versus history of testing well water at least once in the past.

Respondent’s Age	Never (*n* = 374)	Tested at Least Once (*n* = 1028)	Missing (*n* = 324)	Total (*n* = 1726)
18 to 30	26 (19.4%)	56 (41.8%)	52 (38.8%)	134 (100.0%)
31 to 40	110 (21.4%)	298 (57.9%)	107 (20.8%)	515 (100.0%)
41 to 50	147 (22.8%)	398 (61.8%)	99 (15.4%)	644 (100.0%)
51 to 60	61 (22.0%)	172 (62.1%)	44 (15.9%)	277 (100.0%)
61 to 70	19 (19.8%)	65 (67.7%)	12 (12.5%)	96 (100.0%)
71+	9 (16.4%)	37 (67.3%)	9 (16.4%)	55 (100.0%)
Number Missing	2	2	1	5

## Data Availability

The datasets generated and/or analyzed for this study are not publicly available as the datasets contain protected health information. De-identified data may be available upon request from the authors. Requests for research data can be directed to the principal investigator, Dominika Jegen, jegen.dominika@mayo.edu.

## Supplementary Materials

The following supporting information can be downloaded at: https://www.mdpi.com/article/10.3390/ijerph22040504/s1. Supplementary File S1: Patient Survey Questions and Results.

## Abbreviations

The following abbreviations are used in this manuscript:

IQ	Intelligence Quotient
IRB	Institutional Review Board
AIC	Aikaike Information Criterion

## References

[1] Rathi B.S., Kumar P.S., Show P.L. (2021). A review on effective removal of emerging contaminants from aquatic systems: Current trends and scope for further research. J. Hazard. Mater..

[2] Roh T., Knappett P.S.K., Han D., Ludewig G., Kelly K.M., Wang K., Weyer P.J. (2023). Characterization of Arsenic and Atrazine Contaminations in Drinking Water in Iowa: A Public Health Concern. Int. J. Environ. Res. Public Health.

[3] Rogan W.J., Brady M.T., Binns H.J., Forman J.A., Karr C.J., Osterhoudt K., Paulson J.A., Roberts J.R., Sandel M.T., Seltzer J.M. (2009). Drinking water from private wells and risks to children. Pediatrics.

[4] Committee on Environmental Health, Committee on Infectious Diseases (2009). Drinking water from private wells and risks to children. Pediatrics.

[5] Murray C.J., Olson A.L., Palmer E.L., Yang Q., Amos C.I., Johnson D.J., Karagas M.R. (2020). Private well water testing promotion in pediatric preventive care: A randomized intervention study. Prev. Med. Rep..

[6] DeSimone L.A., Hamilton P.A., Gilliom R.J. (2009). Quality of water from domestic wells in principal aquifers of the United States. 1991–2004─Overview of major findings. U.S. Geol. Surv. Circ..

[7] Jegen D., Grygleski M. (2024). Drinking the disease: A family affected by arsenic in well water. Can. J. Rural. Med..

[8] Eaves L.A., Keil A.P., Jukic A.M., Dhingra R., Brooks J.L., Manuck T.A., Rager J.E., Fry R.C. (2023). Toxic metal mixtures in private well water and increased risk for preterm birth in North Carolina. Environ. Health.

[9] Nguyen V.-T., Vo T.-D.-H., Tran T.-D., Nguyen T.-N.-K., Nguyen T.-B., Dang B.-T., Bui X.-T. (2021). Arsenic-contaminated groundwater and its potential health risk: A case study in Long An and Tien Giang provinces of the Mekong Delta, Vietnam. Environ. Sci. Pollut. Res..

[10] Jegen D., Maxson J., Fischer K., Bernard M., Foss R., Hidaka B., Passmore R., Sosso J., Stacey S.K., Thacher T.D. (2024). Arsenic Exposure in Well Water from the Perspective of Patients and Providers. J. Prim. Care Community Health.

[11] Ganie S.Y., Javaid D., Hajam Y.A., Reshi M.S. (2024). Arsenic toxicity: Sources, pathophysiology and mechanism. Toxicol Res..

[12] Agency for Toxic Substances and Disease Registry Toxicological Profile for Arsenic. https://wwwn.cdc.gov/TSP/ToxProfiles/ToxProfiles.aspx?id=22&tid=3.

[13] Jegen D.A., Jannetto P.J. (2023). What’s in your water? A well-known risk for arsenic toxicity. J. Rural. Med..

[14] American Heart Association Chronic Exposure to Lead, Cadmium and Arsenic Increases Risk of Cardiovascular Disease. https://newsroom.heart.org/news/chronic-exposure-to-lead-cadmium-and-arsenic-increases-risk-of-cardiovascular-disease#:~:text=DALLAS%2C%20June%2012%2C%202023%20%E2%80%94,modifiable%20risks%20for%20cardiovascular%20disease.

[15] Sreckovic M., Backovic D., Dugandzija T., Dragicevic I., Nikolic L.P., Mulic M., Damnjanovic B. (2024). Exposure to Arsenic in Drinking Water and Risk of Bladder Cancer. Acta Clin. Croat..

[16] Hsu B.W., Hsiao W.W., Liu C.Y., Tseng V.S., Lee C.H. (2024). Rapid and noninvasive estimation of human arsenic exposure based on 4-photo-set of the hand and foot photos through artificial intelligence. J. Hazard. Mater..

[17] Ahn J., Boroje I.J., Ferdosi H., Kramer Z.J., Lamm S.H. (2020). Prostate Cancer Incidence in U.S. Counties and Low Levels of Arsenic in Drinking Water. Int. J. Environ. Res. Public Health.

[18] Suhl J., Leonard S., Weyer P., Rhoads A., Siega-Riz A.M., Renee Anthony T., Burns T.L., Conway K.M., Langlois P.H., Romitti P.A. (2018). Maternal arsenic exposure and nonsyndromic orofacial clefts. Birth Defects Res. Part. A Clin. Mol. Teratol..

[19] Bulka C.M., Bryan M.S., Lombard M.A., Bartell S.M., Jones D.K., Bradley P.M., Vieira V.M., Silverman D.T., Focazio M., Toccalino P.L. (2022). Arsenic in private well water and birth outcomes in the United States. Environ. Int..

[20] Wei C.F., Mukherjee S.K., Ekramullah S.M., Arman D.M., Islam M.J., Azim M., Rahman A., Rahman M.N., Ziauddin M., Tindula G. (2024). Arsenic modifies the effect of folic acid in spina bifida prevention, a large hospital-based case-control study in Bangladesh. Environ. Health.

[21] Karim M.R., Ahmad S.A. (2014). Nutritional status among the children of age group 5-14 years in selected arsenic exposed and non-exposed areas of Bangladesh. J. Fam. Reprod. Health.

[22] Farzan S.F., Eunus H.M., Haque S.E., Sarwar G., Hasan A.R., Wu F., Islam T., Ahmed A., Shahriar M., Jasmine F. (2022). Arsenic exposure from drinking water and endothelial dysfunction in Bangladeshi adolescents. Environ. Res..

[23] Taylor A., Garretson A., Bieluch K.H., Buckman K.L., Lust H., Bailey C., Farrell A.E., Jackson B.P., Lincoln R., Arneson E. (2024). A Mixed Methods Approach to Understanding the Public Health Impact of a School-Based Citizen Science Program to Reduce Arsenic in Private Well Water. Environ. Health Perspect..

[24] Vahter M., Skroder H., Rahman S.M., Levi M., Hamadani J.D., Kippler M. (2020). Prenatal and childhood arsenic exposure through drinking water and food and cognitive abilities at 10 years of age: A prospective cohort study. Environ. Int..

[25] Wasserman G.A., Liu X., Parvez F., Chen Y., Factor-Litvak P., LoIacono N.J., Levy D., Shahriar H., Uddin M.N., Islam T. (2018). A cross-sectional study of water arsenic exposure and intellectual function in adolescence in Araihazar, Bangladesh. Environ. Int..

[26] Newell M.E., Babbrah A., Aravindan A., Rathnam R., Kiernan R., Driver E.M., Bowes D.A., Halden R.U. (2024). Prevalence rates of neurodegenerative diseases versus human exposures to heavy metals across the United States. Sci. Total Environ..

[27] Wasserman G.A., Liu X., Loiacono N.J., Kline J., Factor-Litvak P., van Geen A., Mey J.L., Levy D., Abramson R., Schwartz A. (2014). A cross-sectional study of well water arsenic and child IQ in Maine schoolchildren. Environ. Health.

[28] Wasserman G.A., Liu X., Parvez F., Factor-Litvak P., Kline J., Siddique A.B., Shahriar H., Uddin M.N., van Geen A., Mey J.L. (2016). Child Intelligence and Reductions in Water Arsenic and Manganese: A Two-Year Follow-up Study in Bangladesh. Environ. Health Perspect..

[29] Criswell R., Gleason K., Abuawad A.K., Karagas M.R., Grene K., Mora A.M., Eskenazi B., Senechal K., Mullin A.M., Rokoff L.B. (2025). A Call for Pediatric Clinicians to Address Environmental Health Concerns in Rural Settings. Pediatr. Clin. N. Am..

[30] Woolf A.D., Stierman B.D., Barnett E.D., Byron L.G., Council on Environmental Health and Climate Change, Committee on Infectious Diseases (2023). Drinking Water From Private Wells and Risks to Children. Pediatrics.

[31] Cradock A.L., Barrett J.L., Nink E., Wilking C. (2024). An economic evaluation of strategies to ensure safer drinking water in the homes of families with young children in select United States locations. Prev. Med. Rep..

[32] Iowa Department of Natural Resources Private Well Program Environmental Protection—Private Water Supply Wells In Iowa; Iowa Department of Natural Resources. https://www.iowadnr.gov.

[33] Minnesota Department of Health Private Well Protection Clean Water Fund. https://www.health.state.mn.us/communities/environment/water/cwf/wells.html.

[34] Wisconsin Department of Natural Resources Wells. https://dnr.wisconsin.gov/topic/Wells.

[35] Minnesota Department of Health Data-Driven Outreach for Private Well Users: Findings from a Statewide Survey on Households on Private Wells with Elevated Levels of Arsenic. https://www.health.state.mn.us/communities/environment/water/docs/cwf/hhsurveyreport.pdf.

[36] Lade G.E., Comito J., Benning J., Kling C., Keiser D. (2024). Improving Private Well Testing Programs: Experimental Evidence from Iowa. Environ. Sci. Technol..

[37] Malecki K.M.C., Schultz A.A., Severtson D.J., Anderson H.A., VanDerslice J.A. (2017). Private-well stewardship among a general population based sample of private well-owners. Sci. Total Environ..

[38] Seliga A., Spayd S.E., Procopio N.A., Flanagan S.V., Gleason J.A. (2022). Evaluating the impact of free private well testing outreach on participants’ private well stewardship in New Jersey. J. Water Health.

[39] Flanagan S.V., Braman S., Puelle R., Gleason J.A., Spayd S.E., Procopio N.A., Prosswimmer G., Navas-Acien A., Graziano J., Chillrud S. (2020). Leveraging Health Care Communication Channels for Environmental Health Outreach in New Jersey. J. Public. Health Manag. Pr..

[40] Argos M., Kalra T., Rathouz P.J., Chen Y., Pierce B., Parvez F., Islam T., Ahmed A., Rakibuz-Zaman M., Hasan R. (2010). Arsenic exposure from drinking water, and all-cause and chronic-disease mortalities in Bangladesh (HEALS): A prospective cohort study. Lancet.

[41] Naujokas M.F., Anderson B., Ahsan H., Aposhian H.V., Graziano J.H., Thompson C., Suk W.A. (2013). The broad scope of health effects from chronic arsenic exposure: Update on a worldwide public health problem. Environ. Health Perspect..

[42] Lampron-Goulet E., Gagnon F., Langlois M.-F. (2017). Association between consumption of private well water contaminated by low levels of arsenic and dysglycemia in a rural region of Quebec, Canada. Environ. Res..

[43] Roh T., Steinmaus C., Marshall G., Ferreccio C., Liaw J., Smith A.H. (2018). Age at Exposure to Arsenic in Water and Mortality 30-40 Years After Exposure Cessation. Am. J. Epidemiol..

[44] Dwivedi S., Mishra S., Kumar V., Agnihotri R., Sharma P., Tiwari R.K., Gupta A., Singh A.P., Kumar S., Sinam G. (2023). A comprehensive review on spatial and temporal variation of arsenic contamination in Ghaghara basin and its relation to probable incremental life time cancer risk in the local population. J. Trace Elem. Med. Biol..

[45] Casalegno C., Candelo E., Santoro G. (2022). Exploring the antecedents of green and sustainable purchase behaviour: A comparison among different generations. Psychol. Mark..

[46] Wang X.W., Wu L. (2024). Intergenerational differences in the environmental concerns of plastic waste business owners: Environmental knowledge, environmental risk exposure, and community connection as mediators. Humanit. Soc. Sci. Commun..

[47] Milfont T.L., Zubielevitch E., Milojev P., Sibley C.G. (2021). Ten-year panel data confirm generation gap but climate beliefs increase at similar rates across ages. Nat. Commun..

[48] Milfont T.L., Osborne D., Sibley C.G. (2022). Socio-political efficacy explains increase in New Zealanders’ pro-environmental attitudes due to COVID-19. J. Environ. Psychol..

[49] Poortinga W., Demski C., Steengjes K. (2023). Generational differences in climate-related beliefs, risk perceptions and emotions in the UK. Commun. Earth Environ..

