# Supplementary material for: Reported Behavioral Patterns and Concern Surrounding Well Water Testing for Arsenic in Midwestern Homeowners with Children
Source: Int J Environ Res Public Health. 2025 Mar 26;22(4):504. doi: 10.3390/ijerph22040504 (PMC12026731; doi:10.3390/ijerph22040504)
Supplement: Supplementary file 1 [file ijerph-22-00504-s001.zip › ijerph-3485556-supplementary.pdf]

International Journal of
Environmental Research and Public Health

MDPI

Supplemental Material - Patient Survey Questions and Results

	Total (*N* = 20,511)
Does your primary home use well water, n (%)	
No	10,840 (53%)
Yes	8994 (44%)
Not sure	316 (2%)
Yes, but did not answer any additional questions	229 (1%)
Missing	132

	Total (N = 8994)
How often do you have your well water tested, n (%)	
Never	1923 (21%)
Once	1415 (16%)
Once a year	1016 (11%)
Every 2 to 5 years	1857 (21%)
Every 6 to 10 years	1214 (14%)
Not sure	1547 (17%)
Missing	22
How often do you worry about the health effects of your well water on you and your family, n (%)	
Never worry about it	3284 (37%)
Rarely worry about it	2931 (33%)
Sometimes worry about it	2057 (23%)
Often worry about it	479 (5%)
Always worry about it	219 (2%)
Missing	24
Do you worry about arsenic in your well water, n (%)	
Never worry about it	5487 (62%)
Rarely worry about it	2068 (23%)
Sometimes worry about it	1028 (12%)
Often worry about it	174 (2%)
Always worry about it	102 (1%)
Missing	135
Would information on how to test your well water for arsenic be useful to you, n (%)	
Not at all useful	856 (10%)
Somewhat useful	2601 (29%)
Very useful	3108 (35%)
Extremely useful	1547 (17%)
Not sure how useful	761 (9%)
Missing	121
What state do you live in, n (%)	
Minnesota	3707 (42%)
Wisconsin	4902 (55%)
Iowa	221 (3%)
Other	27 (0%)
Missing	137
How many children under the age of 18 years currently live in your home, n (%)	
None	7133 (81%)
1 to 2	1297 (15%)
3 to 4	388 (4%)
5 or more	41 (0%)
Missing	135
Current age range, n (%)	

18 to 30 years	340 (4%)
31 to 40 years	625 (7%)
41 to 50 years	1006 (11%)
51 to 60 years	1688 (19%)
61 to 70 years	2826 (32%)
71 years or more	2346 (27%)
Missing	163
What sex do you identify yourself as, n (%)	
Female	5432 (62%)
Male	3266 (37%)
Transgender	7 (0%)
I do not identify as female, male, or transgender	4 (0%)
I do not wish to answer	125 (1%)
Missing	160
Ethnic origin, n (%)	
Hispanic or Latino	64 (0%)
Not Hispanic or Latino	8224 (95%)
I do not wish to answer	367 (4%)
Missing	339
Race*, n (%)	
American Indian/Alaskan Native	85 (1%)
Asian	39 (0%)
Black or African American	24 (0%)
Native Hawaiian or Other Pacific Islander	7 (0%)
White	8430 (94%)
Other	86 (1%)
I do not wish to answer	253 (3%)

*Check all that apply.
